# Longitudinal disturbances of objective sleep architecture in cocaine use disorder: A translational systematic review

**DOI:** 10.1192/j.eurpsy.2026.10488

**Published:** 2026-07-31

**Authors:** I. Medigue, S. Catoire, C. Peyron, P.-A. Geoffroy, T. Bernabeu, L. Peter-Derex, B. Rolland

**Affiliations:** 1 Centre de Recherche en Neuroscience de Lyon (CRNL), Inserm UMR 1028, CNRS UMR 5292, Université Claude Bernard Lyon 1, Université de Lyon, Lyon; 2 Service Universitaire d’Addictologie de Lyon (SUAL), Pôle MOPHA, CH Le Vinatier; 3 Unité Michel Jouvet, 69Z19, Pôle Est, CH Le Vinatier, Bron; 4 Département de Psychiatrie et D′addictologie, AP-HP, GHU Paris Nord, DMU Neurosciences, Hôpital Bichat - Claude Bernard; 5 Centre ChronoS, GHU Paris - Psychiatry & Neurosciences; 6 Université de Paris, NeuroDiderot, Inserm, FHU I2-D2, Paris; 7 Centre de Médecine du Sommeil, Hôpital de la Croix-Rousse, Hospices Civils de Lyon, Université Lyon 1, Lyon, France

## Abstract

**Introduction:**

Cocaine Use Disorder (CUD) is frequently associated with severe and persistent sleep disturbances, affecting both the initiation and maintenance of abstinence. Despite their clinical relevance, the nature, timeline, and underlying neurobiological mechanisms of these disturbances remain insufficiently understood. In particular, objective sleep measures have not been systematically explored across different stages of cocaine use and withdrawal. Improved knowledge of these disruptions is critical for identifying new therapeutic targets and reducing relapse risk.

**Objectives:**

This systematic review aimed to characterize objective alterations in sleep architecture associated with cocaine use and its withdrawal phases. A secondary aim was to compare findings between human and animal studies to identify converging patterns and highlight potential translational mechanisms.

**Methods:**

A systematic search was conducted across PubMed, PsycInfo, and Google Scholar databases, following PRISMA guidelines. Inclusion criteria covered original studies reporting objective sleep data (PSG, EEG, or actigraphy) in the context of cocaine use or withdrawal, in both humans and animal models. Studies involving significant psychiatric comorbidities or other substance use (excluding nicotine) were excluded. Risk of bias was assessed using NIH and ARRIVE tools.

**Results:**

A total of 19 studies were included (12 human, 7 animal). In both populations, cocaine use was associated with reduced total sleep time (TST), decreased sleep efficiency (SE), prolonged sleep onset latency (SOL), and a marked reduction in REM sleep. In early withdrawal, a REM rebound was frequently observed, along with a transient improvement in sleep quantity. However, during later stages of withdrawal, sleep quality deteriorated again, with persistent fragmentation, reduced REM, and prolonged latencies (Image 1). These changes were more consistently captured in human studies. Animal models provided additional insight into underlying mechanisms but showed methodological heterogeneity and limited longitudinal data.

**Image 1:**
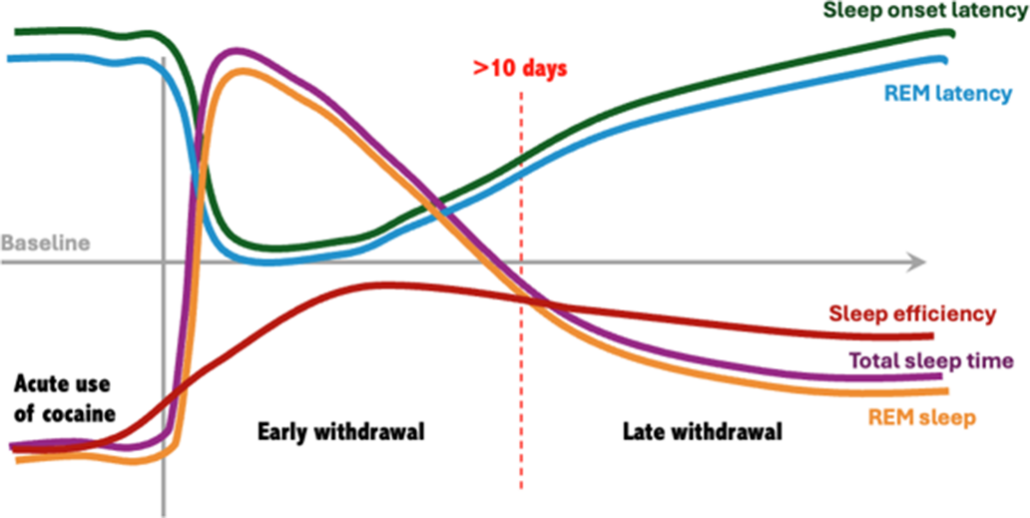
Image 1: Long description.

**Conclusions:**

This is the first systematic and translational review focusing exclusively on objective sleep measures in CUD. Sleep disturbances evolve dynamically across different stages of use and withdrawal, with persistent impairments beyond the acute phase. These findings underscore the importance of integrating sleep assessment into addiction care and suggest that sleep may serve as a clinical target or potential biomarker in the treatment of CUD.

**Disclosure of Interest:**

None Declared

## Long descriptions

### Image 1: Long description

A line graph tracks five metrics across three phases: Acute use of cocaine, Early withdrawal, and Late withdrawal. A horizontal grey line represents the Baseline.

* Phase 1: Acute use of cocaine. Sleep onset latency (dark green) and R E M latency (blue) are high above baseline. Sleep efficiency (red), Total sleep time (purple), and R E M sleep (orange) are well below baseline.

* Phase 2: Early withdrawal. Immediately after use stops, Sleep onset latency and R E M latency drop sharply below baseline. Conversely, Total sleep time and R E M sleep spike sharply above baseline before beginning a steady decline. Sleep efficiency rises toward baseline but remains slightly below it.

* Phase 3: Late withdrawal (marked by a red dashed vertical line at greater than 10 days). Sleep onset latency and R E M latency show a linear increase, moving further above baseline. Total sleep time and R E M sleep continue a steady decline, falling below baseline. Sleep efficiency shows a gradual downward trend further away from the baseline.

Navigate back to Image 1:.

